# When Blood Is Touched

**DOI:** 10.3390/ma2041547

**Published:** 2009-10-16

**Authors:** Leo Vroman

**Affiliations:** 1600 Texas Street, Apt. 1005, Fort Worth, TX 76102, USA; E-Mail: lvroman@flash.net

**Keywords:** blood, surface, interface, biomaterial

## Abstract

The development of blood-compatible materials is reviewed. It grew from originally simplistic views of physical requirements such as surface charge and wettability, to endothelial cells seeded onto a biodegradable cast, and tissue engineering. *In vitro* findings grew from the discovery of one specific protein being adsorbed, to that of sequential protein adsorption with complex implications of platelet and white cell adhesion. The main challenge is still the production of small blood vessels (capillaries).

## 1. The First Events

### 1.1. Early history

The importance of the very first reactions of blood or blood plasma to the touch of a man-made material can be compared to the first few seconds of a traffic accident. These seconds may determine later fatal or non-fatal consequences. Whether drawn into a syringe, placed in a test tube, forced into a maze of membrane or capillary interfaces of a hemodialyzer or a blood pump, to the living blood all these are traffic accidents.

The most ancient observations of surfaces affecting blood clotting probably were made when it was noticed that spilled blood clots faster on wettable surfaces such as glass than on non-wettable ones such as paraffin. More recently, glass tubes coated with silicone and plastic tubes are used to collect the blood in its so-called intact (non-activated) form. 

A simple experiment [1] shows at least two steps are involved in plasma activation. Place intact citrated (to remove calcium which is needed to complete coagulation) plasma into a glass tube and after a few minutes return it to a plastic tube. When calcium is re-added, this sample of plasma will clot in a shorter time than plasma that had never been in contact with glass. So apparently the glass had adsorbed a substance that then became activated and either was released or reacted with another substance in the bulk solution causing further activation. Until about 1963 it was assumed that the first reaction of blood or plasma to contact with a material such as glass was the deposition of this mysterious substance.

One of these substances was a protein called Hageman factor (after the man found to have a long clotting time because he was lacking a newly discovered factor), now called factor XII. 

It seemed that factor XII when adsorbed to glass did become activated and in turn activated another protein, factor XI, which then was liberated in active form, all before calcium had been re-added [2].

I was fortunate enough to obtain an ellipsometer (an instrument sensitive to less than a few Angstroms of adsorption at an interface), and we found that indeed, something was rapidly adsorbed out of plasma and then, slowly, and if the plasma was not missing factor XII, something was desorbed [3]. I assumed of course that I had seen the adsorption of something including factor XII which then had adsorbed factor XI and released it. I proved wrong.

### 1.2. The intrinsic versus extrinsic clotting systems

Before the serious search for biomaterials that could be compatible with blood (not activating it), the intrinsic clotting system, so called because the blood itself contained all the necessary proteins to cause clotting, seemed of no clinical importance. It is a slow system, requiring about 6 minutes to complete. The extrinsic system is one that needs a substance from the cut tissue of a wound, which makes the blood clot in less than 30 seconds and helps the victim to stop bleeding. Only with inventions such as dialyzers and the search for materials to fabricate blood vessels, all requiring contact with blood without wounds, did the intrinsic system become clinically important. 

### 1.3. Earliest contact—General

Onto wettable materials, most proteins are readily adsorbed and will cause the surface to become less hydrophilic [4], possibly by spreading and exposing some hydrophobic amino acid residues. Onto hydrophobic surfaces, the proteins most ready to expose hydrophobic amino acid residues by changing their structure will be the ones most readily adsorbed [5].

We, and others [6,7] found that there is a sequence of proteins being deposited of which each one is replaced by the next. Among those we studied, this sequence was: albumin, immunoglobulins (IgG), fibrinogen [8], factor XII and when high molecular weight kininogen (HMWK) was discovered, it proved the agent that displaced fibrinogen [9,10]. 

### 1.4. Factors affecting rate and extent of these events

#### 1.4.1. Chemical composition of the substrate

Following the early theory of Sawyer [11] and others that a negative surface charge would emulate that on the surface of blood vessels and should therefore be non-thrombogenic, we were asked to study those with the most negatively charged down to the most reasonable metals in our ellipsometer. We found magnesium to become coated with a thick layer of oxide even before it could do anything else, and aluminum to become oxidized while adsorbing proteins, so we abandoned this effort. Connecting a metal substrate to the negative pole of a power supply also was non-informative. Among a large variety of materials submitted to us in contracts with the National Institute of Arthritis and Metabolic Diseases, we found marked differences; unfortunately, we were not allowed to disclose them. On the hydrophobic surfaces we studied, the protein sequence appeared to stop after deposition of fibrinogen, and gamma globulins behaved quite differently from that on hydrophilic surfaces [12].

#### 1.4.2. Physical circumstances

##### Flow

**Figure 1:**
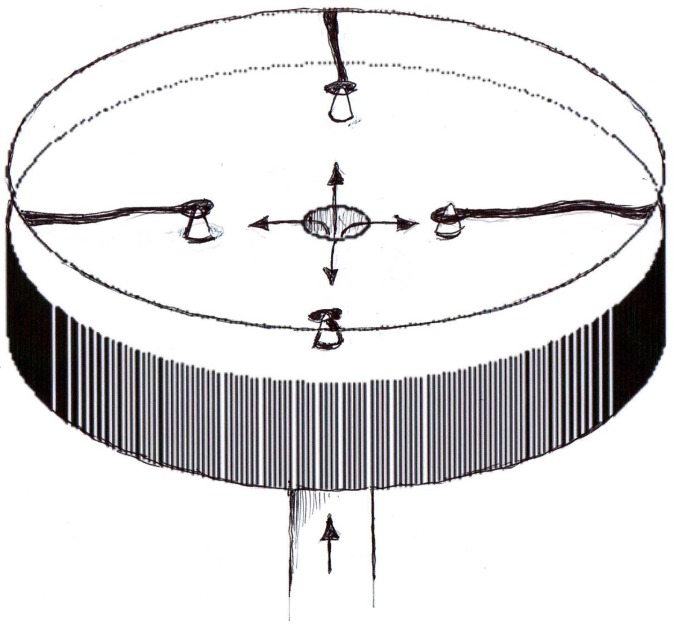
Diagram of stagnation point flow chamber, showing coverslip supported by 4 points. Arrows indicate flow. Areas where flowing plasma had left fibrinogen on the coverslip are shown in black.

Hoping to find a difference between events at the stagnation point of flow impinging vertically onto a glass surface, we obtained 2 stagnation point flow chambers. We passed diluted intact plasma through them and found 3 surprises: the stagnation point showed no difference with its near periphery, but in the wake of disturbed flow beyond the chamber’s small support points that separated the glass coverslip from the chamber floor, fibrinogen was much more slowly removed. Also, in the narrow spaces close around these points, fibrinogen remained longer [13] (see Figure 1). 

More precise studies have been done on effects of separated flow [14,15] where, in the shelter of a step, fibrinogen remained adsorbed longer.

##### Space width

**Figure 2:**
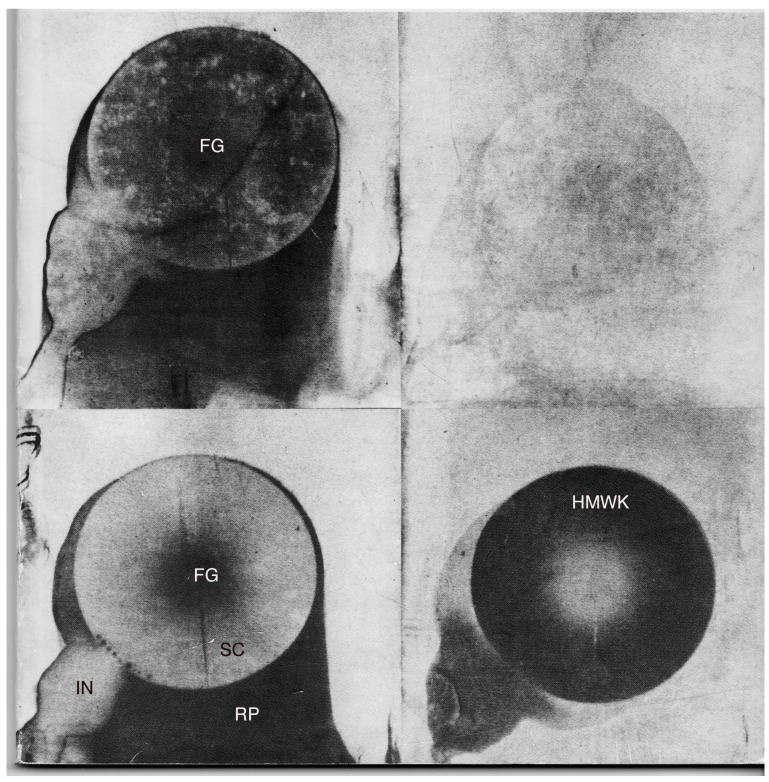
Undiluted intact citrated plasma from a patient lacking HMWK was injected between a lens, curvature 147 mm., and a glass slide. After 10 min. the slide was rinsed, exposed to antiserum to fibrinogen (left set) or to HMWK (right set). The bottom two images were obtained by adding 1.4 U of purified HMWK to 1 ml of the patient’s plasma before the experiment. J. Biomed. Mater. Res. Vol. 18, 1984, 6643-6654 [16]. Copyright 1984, John Wiley & Sons, Inc., modified with permission of John Wiley & Sons, Inc.

Our findings with the stagnation point flow chamber led us to investigate the effect of narrow spaces more systematically. In a series of experiments, we placed a convex lens (preferred radius of curvature about 147 mm) on a glass or anodized tantalum coated glass slide and injected diluted intact plasma between lens and slide. After ten minutes, we tilted the slide and rinsed the lens off the slide with normal saline or veronal buffer, then covered it with the proper antiserum. After about 4 minutes the slides were rinsed again with saline or buffer, then water, and glass slides were then stained with Coomassie Blue, while the anodized tantalum ones were dried and simply observed for change in interference color [16] (see Figure 2). 

FG fibrinogen left in the narrow space at center; IN site of injection of the plasma; RP fibrinogen left during rapid rinsing; SC scratch left by the lens being rinsed away, where plasma had time to refill it with fibrinogen.

In general, concentric circles of proteins are left on the slide, depending among others on the dilution of intact plasma used, with albumin surrounded by IgG surrounded by fibrinogen surrounded by HMWK. The border between each two proteins is most probably caused by the critical amount of the displacing protein present in the column of plasma over the site [17]. Using a lens-on-slide system combined with ellipsometry, it was found that on vanadium and on silver, fibrinogen was not removed [17].

##### Surface energy

Most findings described above refer to wettable surfaces such as those of glass or anodized metals all having a high critical surface tension. It has been assumed by some [18] that there is an optimal region of this value that prevents thrombosis *in vitro* and should be aimed for in fabricating blood compatible biomaterials. In contrast to our findings that small differences in surface charge had little effect, the extreme difference between wettability and non-wettability was dramatic. For example, while I found many hydrophilic insoluble powders remove prothrombin and factor IX from plasma, a hydrophobic powder (barium stearate) removed factor V predominantly [19]. I assumed it was adsorbed most of all clotting factors because it most readily exposed its hydrophobic amino acid residues [20], which agrees with the description of “soft” proteins and their tendency to be adsorbed onto hydrophobic surfaces [5]. In general, we found that the sequence of proteins displacing each other on a hydrophobic surface stops at fibrinogen. 

## 2. Subsequent Events

### 2.1. Platelets

Blood platelets are like small cells without a nucleus; they may be regarded as alive as are white blood cells. They aggregate at wounds, sealing them while promoting the formation of a well organized blood clot, but if stimulated to aggregate inside a vein, will cause some formation of fibrin clot downstream: a thrombus. We found that *in vitro*, platelets adhere to surfaces where fibrinogen is adsorbed [21,13]. Consequently, on glass, where upon contact with intact normal human blood, fibrinogen will arrive within about 3 seconds and is replaced by HMWK within 30 seconds, platelets arrive too late to adhere, while onto hydrophobic surfaces, where fibrinogen lingers, they will adhere. They do so with specific receptors that recognize an area on the fibrinogen molecule [22], and should not be regarded as physically more or less sticky. Any test previously regarded as measuring stickiness actually measures the time fibrinogen has remained on the test surface.

### 2.2. White blood cells

We found that granulocytes adhered to immunoglobulin IgG where this protein had been pre-adsorbed onto a hydrophobic rather than a hydrophilic substrate [23]. However, this action of IgG may be indirect: we found for example that normal activated plasma deposited a large amount of matter onto pre-adsorbed 7s gamma-globulin [24]. The ability of polymerized IgG to activate the complement system is well known [25], and Craddock *et al*. found that as a result, granulocytes become lost during dialysis with membranes of certain materials [26].

## 3. Theoretical Approaches 

I found five possible explanations for the sequential adsorption of proteins out of plasma:

Horbett [7] assumes mass action (protein concentration) is responsible.

Willems *et al.* [27] calculate that protein mobility and lateral interactions at the interface may be mainly involved in the unexpectedly steep drop of adsorption rate with increased adsorption, as well as in the so-called Vroman effect (sequential adsorption). Van der Waals forces would be involved at close contact.

Noh and Vogler [28] calculate that molecular size may be the responsible factor.

Lu *et al.* [29] compute that interactions between diffusive-convective protein transport and competitive adsorption and displacement kinetics are responsible but require near normal concentrations of each protein, in diluted plasma.

LeDuc *et al.* [30] generalize the usual Langmuirian formulation to apply fitting parameters for each protein.

I find it striking that both theories: the one based on protein concentration and the one based on protein molecular size, appear at least partly applicable. It implies that there is a remarkable correlation between the two: our plasma contains proteins whose concentration decreases with increasing molecular weight, in general. 

One possibility that I considered is that any adsorbed protein species can only be removed by a molecule of the same species, forming a temporary dimer that forces the adsorbed molecule back into its original conformation and into solution. Brash [31] did show that adsorbed fibrinogen exchanges with fibrinogen in solution.

There are two aspects to these studies that I feel need consideration.

(1) It may seem miraculous that my blood has been circulating in my body for more than 94 years without noticeable mishap. However, this physiological miracle has taken millions of years to perform and is therefore not a miracle, but evolution. The blood has evolved allowing us to survive by reacting to infections and to any other small invasions. Removing a drop of my blood from its blood vessel wall environment and placing it on a slide, I will see my intrinsic coagulation system and my platelets waking up to seal a wound that does not exist, and through a microscope I can see granulocytes spreading on the glass surface and crawling for an hour or so trying to eat the slide and in my absence protect me from an invasion of glass. Hoping to get a clearer insight in blood by isolating it is like cutting off a limb to study its essential functions.

(2) If we must really implicate van der Waals forces, we will unavoidably descend into electron behavior and possibly quantum physics, where problems of protein behavior could only be computed but never quite understood. Applying such findings to the total plasma and blood will be impossible. There is a great gap from molecule to me:

 

Could I, trying to understand,

reach across that giant gap

and place with a space-engulfing hand

a protein molecule in my lap,

to hold it near and even nearer

to see her shivering within

her quaint quantum mechanical skin—

would she be any clearer?

 

Put that molecule into a cell

and that cell will have to balloon

becoming as big, and as far as well,

as the moon.

 

Put that cell in my pituitary gland

and that grows as big as the sun …

 

and I will be left to understand

even less by what I’ve done.

 

## 4. The Other Solutions

Instead of basing the choice of material on its simple properties, the search for non-thrombogenicity has become more physiological.

### 4.1. Coatings

#### 4.1.1. Albumin 

With increased residence time on a surface, a protein (at least fibrinogen) will become less elutable [32]. We found that fibrinogen residing briefly on glass could be replaced by intact normal plasma, but not fibrinogen that had resided there 10 minutes or more (unpublished data). Somewhat prolonged exposure of a very wide range of materials to albumin was expected to provide a durable inhibition of further protein interactions and platelet adhesion at the surface [33,34]. 

#### 4.1.2. Heparin

Among various other molecules, heparin takes a special place. Its use began with Vincent Gott’s triple serendipity [35,36,37]: on the basis of Sawyer’s view that a negative charge provided antithrombogenicity [11], Gott obtained graphite surfaces which he connected to the negative pole of a power supply. They performed rather well, but did equally well when the connection to the supply was broken. He found that the graphite had adsorbed the benzalkonium chloride used for sterilization and that it in turn had bound heparin. The relative success of heparin binding has recently been reviewed [39]. The coating may be released into solution [38].

#### 4.1.3. Chitosan 

Poly(*N*-acetyl-D-glucosamine) is one of many other substrates tested [40].

### 4.2. Building live vessels: Tissue engineering

The modern trend in creating capillaries and small veins, being the most difficult of all vessels to keep from being closed by thrombi, is to remove the material from the biomaterial, by seeding endothelial cells [41,42,43,44] preferably on a biodegradable mold. That still would not reproduce the contractility of the vessels. With one step closer to imitating live tissue, endothelial cells are allowed to be seeded onto material that used to be alive, such as pig valves that have been fixed with glutaraldehyde [45] and from which the original cells have been removed [46]. Short of transplantation, the field of tissue engineering has expanded sufficiently to deserve its own journal.

## 5. Conclusions

Many materials, each with many surface treatments and several choices of coating substances, and subsequent *in vitro* and *in vivo* methods of testing the results, all combine to an overwhelming number of approaches to blood compatibility, especially in the production of venules and capillaries. The most physiological approaches in tissue engineering, by not trying to emulate only a number of known properties of blood vessels, but aiming for creation of live structures, may be most hopeful.
